# Subclinical cardiovascular risk signs in adults with juvenile idiopathic arthritis in sustained remission

**DOI:** 10.1186/s12969-020-00448-3

**Published:** 2020-07-14

**Authors:** Inmaculada Concepción Aranda-Valera, Iván Arias de la Rosa, Rosa Roldán-Molina, María del Carmen Ábalos-Aguilera, Carmen Torres-Granados, Alejandra Patiño-Trives, María Luque-Tevar, Alejandro Ibáñez-Costa, Rocío Guzmán-Ruiz, María del Mar Malagón, Alejandro Escudero-Contreras, Chary López-Pedrera, Eduardo Collantes-Estévez, Nuria Barbarroja

**Affiliations:** 1 Rheumatology Department, IMIBIC/Reina Sofía University Hospital/University of Cordoba, Cordoba, Spain; 2 Medicine Department, University of Cordoba/IMIBIC/Reina Sofía University Hospital, Cordoba, Spain; 3 Department of Cell Biology, Physiology and Immunology, IMIBIC, Reina Sofía University Hospital, University of Córdoba, Cordoba, Spain; 4 CIBER Fisiopatología de la Obesidad y Nutrición (CIBEROBN), Instituto de Salud Carlos III, Madrid, Spain

**Keywords:** Juvenile idiopathic arthritis, Cardiovascular risk, Clinical remission

## Abstract

**Background:**

Juvenile Idiopathic Arthritis (JIA) is one of the most common chronic diseases of childhood that often persists into adulthood and can result in significant long-term morbidity. As a long lasting chronic inflammatory disease, concern has been raised regarding the risk of premature development of cardiovascular disease (CVD) in JIA. This study aims to determine whether adults with JIA in clinical remission display clinical and subclinical signs of CVD risk: inflammatory mediators, adipokines, endothelial dysfunction and oxidative stress markers.

**Methods:**

This is a cross-sectional study including 25 patients diagnosed with JIA according to the International League of Associations for Rheumatology criteria (ILAR 2001) and 25 age- and sex-matched controls. Remission was determined by JADAS10 < 1 and according to Wallace criteria. The presence of traditional CVD risk factors was analyzed. An extensive clinical analysis including body mass index (BMI), lipid profile, homeostatic model assessment – insulin resistance (HOMA-IR) and arterial blood pressure was performed. Intima media thickness of the common carotid artery (CIMT) was measured as a marker of subclinical atherosclerosis. Several proinflammatory cytokines, molecules involved in the endothelial dysfunction, oxidative stress and adipokines were quantified on serum by ELISA and on peripheral blood mononuclear cells (PBMCs) by RT-PCR. In vitro studies were carried out in healthy PBMCs, adipocytes and endothelial cells which were treated with serum from JIA patients under sustained remission.

**Results:**

Mean duration of the disease was 13.47 ± 5.47 years. Mean age was 25.11 ± 7.21. Time in remission was 3.52 ± 3.33 years. Patients were in remission with no treatment (40%) and with treatments (60%). CVD risk factors and CIMT were similar in JIA patients and controls. However, cholesterol levels were significantly elevated in JIA patients. Levels of adipocytokines, oxidative stress and endothelial activation markers were elevated in serum and PBMCs from JIA patients. Serum of those JIA patients induced the activation of adipocytes, endothelial cells and healthy PBMCs.

**Conclusions:**

JIA adult patients in remission have subclinical signs of inflammation and CVD risk, showed by an increase in the levels of inflammatory cytokines, endothelial activation and oxidative stress markers and adipokines, molecules closely involved in the alteration of the vascular system.

## Background

Juvenile idiopathic arthritis (JIA) is the most common chronic inflammatory arthritis in children and young people, with onset under the age of 16 years and being characterized by long standing pain, swelling and stiffness in joints [1, 2]. In the pathogenesis and progression of JIA, the unbalance between pro- and anti- inflammatory cytokines might be involved in the regulation of systemic inflammation, local joints damage and bone erosion [3]. In recent decades, there has been considerable interest in the long-term outcomes of individuals with chronic inflammatory arthritis and an area of particular concern has been the increased prevalence of cardiovascular disease (CVD). This increased risk is attributed to a higher prevalence of traditional CVD risk factors and the role of systemic inflammation in the acceleration of atherosclerosis. In fact, increased cardiovascular mortality and morbidity have been observed in Rheumatoid arthritis (RA) patients [4].

Previous studies found increased traditional CVD risk factors in JIA, including family history of CVD, hypertension, and even smoking habit [5]. Additionally, an alteration of the lipid profile has been observed. Although that data could be controversial, most of the studies agree with the elevation of the levels of low density lipoproteins (LDL) and triglycerides and the decrease of high density lipoproteins (HDL) levels [5]. One of the best validated methods to evaluate CVD risk is the pathological increase in the intima media thickness (CIMT), which predicts the early atherogenesis and thus the occurrence of future cardiovascular events in the general population [6]. Hence, in other autoinflammatory diseases such as RA, the pathological increase in CIMT has been related to the enhanced risk of suffering cardiovascular events [7–9].

The chronic inflammation is also a well-defined nontraditional CVD risk in the pathogenesis of atherosclerosis [10], where cytokines including IL-1, IL-6 and TNF-α could promote endothelial dysfunction, a key process in atherogenesis [11, 12]. Few studies have evaluated the CIMT in JIA patients showing controversial data [13–15]. The most recent results point out to an increase in CIMT of these patients, correlating with endothelial dysfunction parameters [16].

Others subclinical CVD risk signs have been identified in the general population. Among them, in JIA, circulating levels of intercellular adhesion molecule (ICAM) and E-selectin are elevated [17]. On the other hand, adipokines, cytokines released mainly by the adipose tissue, are key factors in the metabolic comorbidities that increase the CVD risk. Not only contribute to the regulation of process mediated by insulin, glucose and lipid metabolism, vascular changes and coagulation, but also participate in the chronic inflammatory state. Thus, in severe JIA, levels of leptin are elevated regardless the fat mass [18]. However, to date, few studies have evaluated the role of adipokines in the pathogenesis of JIA.

Diverse studies have suggested that JIA is associated with an increased CVD risk, due to a higher prevalence of traditional CVD risk factors and the cumulative damage from chronic inflammation. Most of these works have been carried out in patients with acute phase. Beyond to an active state, it is unknown whether inflammatory mediators, molecules released by adipose tissue and endothelial dysfunction markers could be present in JIA in sustained remission, contributing to the cardiovascular risk associated with this disease.

Our study provides new evidences about subclinical cardiovascular risk signs that could be present in patients with a remission state of the disease, which could alert the clinical specialist about the need for a tighter control of the disease.

## Methods

### Patients

Twenty-five JIA patients and twenty-five healthy donors (HDs) age-sex-matched with no history of autoimmune disease were included in this cross-sectional study. The participants were Caucasian and recruited at Rheumatology Department (pediatric unit), Reina Sofia University Hospital, Cordoba, Spain, after approval from the ethics committee and signed the informed consent.

The recruitment participant’s diagnoses were made according to the International League of Associations for Rheumatology (ILAR) criteria [19]. Disease activity was assessed using the Juvenile Arthritis Disease Activity Score-10 (JADAS-10), C reactive protein (CRP) [20] considering states of inactive disease, cutoff values ≤1 [21]. Remission state was determined according to Wallace criteria [22]. Regarding treatment regimen, 40% out of patients were in remission with no treatment (*n* = 10) and 60% were under treatments (*n* = 15) (Table 1). Four patients were on minimal doses of prednisone as maintenance treatment (maximum 5 mg / day) at the time of the recruitment.

**Table 1:** Clinical details of JIA patients and healthy donors

Clinical parameters	Healthy donors ***n*** = 25	JIA patients ***n*** = 25	***p*** value
Female/Male (n/n)	13/12	14/11	
Age (years)	27.21 ± 2.54	25.11 ± 7.21	
Disease duration (years)	–	13.47 ± 5.47	
Remission duration (years)	–	3.52 ± 3.33	
RF + (n)	0	1	
ACPAs + (n)	0	1	
CRP (mg/dl)	0.95 ± 1.19	2.88 ± 5.54	
ESR (mm/h)	6.53 ± 3.82	5.47 ± 3.54	
C3 (mg/dL)	124.15 ± 12.51	124.16 ± 16.03	
C4 (mg/dL)	20.84 ± 4.01	25.97 ± 7.19	0.017
**JIA subtypes**			
Systemic (%)	–	4	
Oligoarthritis (%)	–	24	
RF-negative polyarthritis (%)	–	20	
RF-positive polyarthritis (%)	–	4	
Psoriatic (%)	–	24	
Enthesitis-related arthritis (%)	–	24	
Undifferentiated (%)	–	0	
**Metabolic profile**			
BMI	22.49 ± 3.13	22.70 ± 4.32	
Glucose (mg/dl)	83.64 ± 6.38	76.47 ± 11.20	
Insulin (mU/L)	7.10 ± 3.52	5.79 ± 2.76	
HbA1c (%)	5.12 ± 0.11	5.15 ± 0.30	
Total Cholesterol (mg/dl)	163.57 ± 24.75	180.26 ± 29.92	0.046
HDL-Cholesterol (mg/dl)	52.21 ± 10.23	57.63 ± 15.22	
LDL-Cholesterol (mg/dl)	95.50 ± 21.46	105.00 ± 29.17	
Triglycerides (mg/dl)	75.35 ± 33.87	83.31 ± 50.58	
ApoA (mg/dl)	136.35 ± 25.50	140.77 ± 24.16	
ApoB (mg/dl)	69.64 ± 15.63	77.11 ± 18.36	
**Treatments**			
No treatment (n)	–	10	
Corticosteroids (n)	–	4	
Salazopyrin (n)	–	2	
NSAIDS (n)	–	3	
Methotrexate (n)	–	2	
Anti-TNF-α (n)	–	4	

Values are means ± SD, unless otherwise stated

JIA, juvenile idiopathic arthritis; RF, rheumatoid factor; ACPAs, antibodies to citrullinated protein antigens; JADAS: juvenile idiopathic arthritis disease activity score; CRP, C-reactive protein; ESR, erythrocyte sedimentation rate; C3, complement component 3; C4, complement component 4; BMI, body mass index, HbA1c, hemoglobin A1c; HDL, high density lipoprotein; LDL, low density lipoprotein; ApoA, apolipoprotein A; ApoB, apolipoprotein B; NSAIDS, non-esteroidal anti-inflammatory drugs; TNF-α, tumor necrosis factor alpha

Peripheral blood samples were collected from patients and HDs following fasting for 8 h for laboratory tests. Tests were performed in all patients to determine the presence of anti-citrullinated protein antibodies (ACPAs) and rheumatoid factor (RF). Besides, metabolic profile such as, glucose, insulin, hemoglobin 1Ac, total cholesterol (TC), HDL-cholesterol, LDL-cholesterol, triglycerides (TGs), apolipoprotein A (ApoA), apolipoprotein B (ApoB), acute phase reactants such as CRP and erythrocyte sedimentation rate (ESR) and complement factors as complement component 3 (C3) and component 4 (C4) were recorded (Table 1). Additionally, the presence of traditional cardiovascular risk factors including smoking, obesity based on body mass index (BMI > 30 Kg/m2), type 2 diabetes mellitus (T2DM) (fasting blood glucose levels > 126 mg/dL, hemoglobin A1c level > 6.5% or antidiabetic treatment) and hypertension were analyzed. Likewise, the prevalence of Metabolic Syndrome (MetSyn) was evaluated according to the National Cholesterol Education Program (NCEP) adult treatment panel III (ATP III) criteria, where 3 of the 5 following characteristics are met: abdominal obesity (male (> 102 cm); female (> 88 cm), TG > 150 mg/dL, HDL cholesterol (male (< 40 mg/dL); female (< 50 mg/dL); blood pressure > 130/85 mmHg; fasting glucose > 110 mg/dL).

### Carotid intima media thickness

All subjects underwent high-resolution B-mode ultrasonography for carotid intima media thickness (CIMT) measurements. All ultrasound scanning was performed by a single experienced vascular sonographer on the left and right common carotid arteries, using carotid duplex equipment (LOGIC E9). IMT was measured at the distal wall of the carotid artery on a 10-mm segment and defined as the distance from the leading edge of the lumen-intima surface to the leading edge of the media–adventitia interface of the far wall.

### Atherogenic risk

Atherogenic risk was calculated by atherogenic index (AI) based on the levels of TC (mg/dL) and HDL (mg/dL): AI = TC / HDL. Risk was delimited as > 4.5 in female and > 5 in male [23].

### Apolipoprotein B/A risk

Apolipoproteins ratio was used to establish CVD risk due to the levels of apolipoproteins A and B. Relative CVD risk groups were: low CVD risk (female: 0.30–0.59; male: 0.40–0.69), moderate CVD risk (female: 0.60–0.79; male: 0.70–0.89) and high CVD risk (female: 0.8–1.0; male: 0.9–1.1). In this study, to calculate the prevalence of CVD risk by ApoB/ApoA ratio, subjects were separated in two groups: low CVD risk and moderate-high CVD risk [24, 25].

### SCORE CVD risk

SCORE model was used to determined CVD risk based on traditional CV risk factors such as sex, age, systolic pressure, smoking, TC and HDL-cholesterol following EULAR recommendations for CVD risk [26].

### Insulin resistance

The homeostatic model assessment-insulin resistance (HOMA-IR) index was used to measure IR: [insulin concentration (mU/L) x glucose concentration (mg/dL)]/405 (HOMA-IR values > 2.5 indicated IR) [27].

### Serum levels of adipokines, cytokines and adhesion molecules

Serum levels of tumor necrosis factor-alpha (TNF-α), interleukin 1-beta (IL-1β), interleukin 6 (IL-6), intercellular adhesion molecule (ICAM-1), E-selectin and vascular endothelial growth factor A (VEGF-A) were quantified by enzyme-linked immunosorbent assay, following the manufacturer’s instructions (Bionova, Diaclone, Madrid, Spain). Serum levels of leptin, adiponectin (adipoQ), resistin (Bionova, Cusabio, Madrid, Spain) and visfatin (RayBiotech, Norcross, GA (EEUU)) were determined by ELISA following the manufacturer’s instructions.

### Peripheral blood mononuclear cells (PBMCs)

PBMCs were isolated from JIA patients and HDs through Ficoll density gradient. Total RNA was extracted from PBMCs by using a RNA purification kit following the manufacturer’s instructions (Norgen Biotek Corp., ON, Canada). The RNA purity was verified by optical density (OD) absorption ratio OD260/OD280 between 1.8 and 2.0.

### In vitro studies

Mechanistic studies were performed with PBMCs isolated from HDs and cell lines: human umbilical vein endothelial cells (HUVEC) and 3 T3-L1 adipocytes (murine).

Isolated PBMCs from HDs were cultured in medium [RPMI 1640 containing 2 mM L-glutamine, 100 U/mL penicillin, 100 mg/mL streptomycin and 250 pg/mL fungizone (BioWhittaker/MA Bioproducts, Walkersville, MD, USA) at 37 °C in a humidified 5% carbon dioxide (CO2)] atmosphere and treated with 10% of inactivated serum (incubated at 56% for 30 mins) from JIA patients and HDs for 24 h. Cells were collected for RNA isolation and applied to RT-PCR analysis.

HUVEC cells were purchased from ATCC (Manasas, VA, USA). Cells were cultured in Endothelial Cell Basal medium (EBM; Lonza, Walkersville, Md) with 10% FBS, 0.1% human epidermal growth factor (hEGF), 0.1% hydrocortisone, 0.1% gentamicin, amphotericin-B (GA-1000), 0.4% bovine brain extract, 100 U/mL penicillin, 100 mg/mL streptomycin, and 250 pg/mL fungizone (BioWhittaker/MA Bioproducts, Walkesvile, Md) at 37 °C in a humified 5% CO2 atmosphere. For in vitro experiments, HUVECs were seeded into 6-well plates (4 × 10^5^ cells per well) in 1.5 mL of completed medium. After 24 h, cells were treated with 10% of inactivated serum from JIA patients and HDs for 24 h. Subsequently, cells were collected for mRNA analyses.

3 T3-L1 cells were purchased from ATCC (Manasas, VA, USA). Cells were cultured and differentiated into adipocytes according to the protocol described previously [28]. Differentiated cells were used when at least 90% showed an adipocyte phenotype by accumulation of lipid droplets by day 8. On day 8 of differentiation, cells were treated with medium containing 10% of inactivated JIA or HDs serum for 24 h. Cells were harvested for total RNA isolation and applied to gene expression studies.

### Gene expression analysis

The expression of genes involved in inflammation (TNF-α, IL-1β IL-6, IL-8, interferon (IFN)-γ monocyte chemoattractant protein-1 (MCP-1), toll like receptor (TLR)-2 and TLR-4), oxidative stress (superoxide dismutase (SOD)-1, SOD-2, inducible nitric oxide synthase (iNOS), endothelial nitric oxide synthase (eNOS) and glutathione peroxidase (GPX)-1, endothelial dysfunction (VEGF-A, ICAM-1, vascular cell adhesion molecule (VCAM)-1 and E-selectin) and adipokines (leptin, adiponectin, visfatin and resistin) was analyzed in PBMCs, endothelial cells and adipocytes through RT-PCR.

Real-time PCR using SYBR green was performed according to the manufacturer’s instructions (Thermo Fisher Scientific, Madrid, Spain). Expression of genes of interest was corrected by the geometrical average of β-actin, glyceraldehyde-3-phosphate dehydrogenase and hypoxanthine-guanine phosphoribosyltransferase using the BestKeeper tool [29].

### Statistical analysis

Statistical analysis and graphs were performed by GraphPad Prism 8.0.1. Descriptive data are presented as the mean and standard deviation (SD) for continuous variables and as absolute frequencies and percentages for categorical ones. Kolmogorov-Smirnov test for normal distribution was carried out. To compare two independent groups (HDs vs JIA patients, JIA patients with treatments vs JIA patients with no treatment, cells treated with HD serum vs cells treated with JIA serum), following normality and equal variance tests, Student’s t-test or alternatively a non-parametric test (Mann-Whitney rank sum test) were used. Qualitative data was analyze using Chi-squared test (presence of cardiovascular risk factors). Correlations among the levels of serum adipocytokines, lipid profile and clinical parameters were assessed by Spearman’s rank correlation. *p* < 0.05 was considered statistically significant.

Multiple testing correction with fold discovery rate approach was carried out in all the in vivo measures. In these cases, statistical significance was considered when *p* value< 0.05 and FDR < 0.1.

## Results

### Clinical inflammatory markers and cardiovascular risk factors in JIA patients in remission

In our cohort of JIA patients, mean age was 25.11 ± 7.21 years old, with a mean duration of the disease of 13.47 ± 5.47 years. Most of them were in clinical remission for more than 2 years, with mean remission time of 3.52 ± 3.33 years. Regarding lipid profile, total cholesterol levels were significantly increased in JIA patients compared to healthy donors. Of note, although these patients were in remission, levels C4 were significantly elevated (Table 1).

**Fig. 1:**
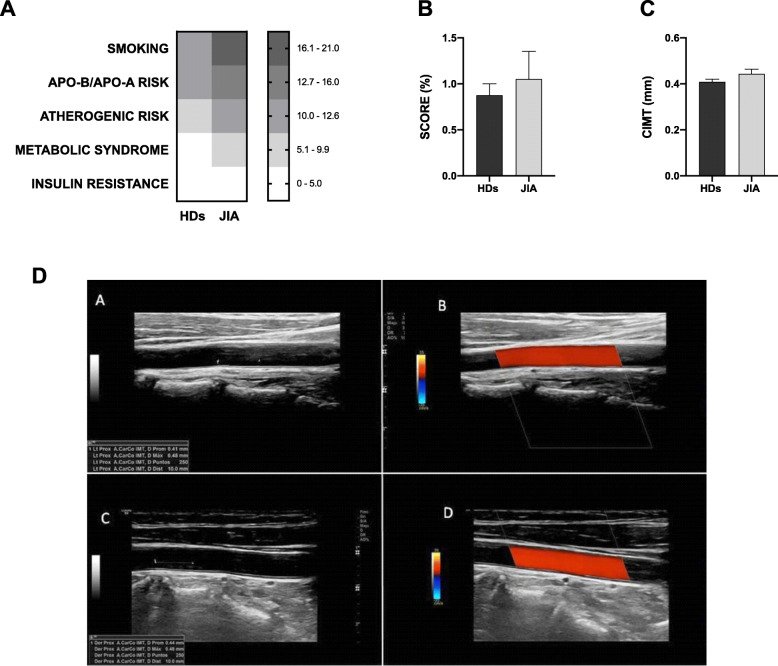
CVD risk factors and subclinical atherosclerosis in JIA patients in remission. **a** Heatmap of CVD risk factors between JIA patients and HDs. Data expressed in percentage (%). **b** SCORE CVD risk in JIA patients compared to HDs. **c** CIMT in JIA patients compared to HDs. **d a**: HD greyscale cIMT; **b**: HDs Power Doppler cIMT; **c**: JIA greyscale cIMT; **d**: JIA Power Doppler cIMT. CVD, cardiovascular disease; JIA, juvenile idiopathic arthritis; HDs, Healthy donors

Traditional CVD risk factors such as smoking and metabolic syndrome were elevated in JIA compared to age-gender matched HDs. Increased ApoB/ApoA and atherogenic risks were also increased in JIA patients, although there were no statistically differences in all these parameters (Fig. 1a). Regarding insulin resistance and BMI, no changes in the rates between patients and healthy donors were noticed (Fig. 1a and Table 1).

SCORE was similar in JIA patients and HDs (Fig. 1b). Likewise, there was no significant difference in the levels of CIMT between JIA patients and HDs (0.44 ± 0.009 vs 0.41 ± 0.017, *p* = 0.078) (Fig. 1c).

### Serum levels of adipocytokines and endothelial adhesion molecules in JIA patients in remission

**Fig. 2:**
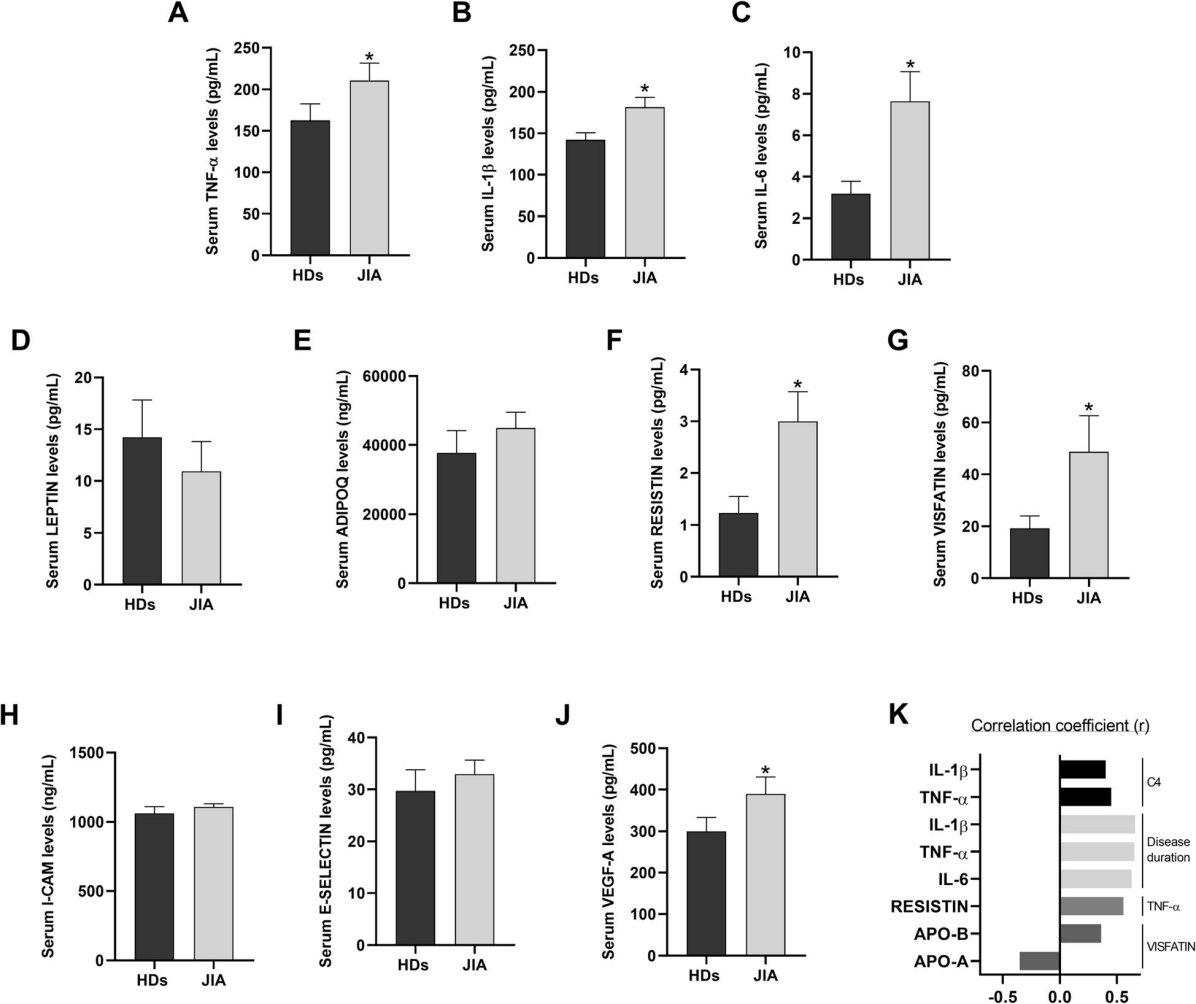
Adipocytokines and endothelial adhesion molecules in serum of JIA patients in remission compared to HDs. **a** Serum TNF-α levels. **b** Serum IL-1β levels. **c** Serum IL-6 levels. **d** Serum Leptin levels. **e** Serum AdipoQ levels. **f** Serum Resistin levels. **g** Serum Visfatin levels. **h** Serum ICAM-1 levels. **i** Serum E-Selectin levels. **j** Serum VEGF-A levels. **k** Correlation coefficients of circulating molecules and clinical parameters. TNF-α: tumor necrosis factor-alpha; IL-1β: interleukin-1β; IL-6: interleukin-6; AdipoQ: adiponectin; VEGF-**a**: vascular endothelial growth factor-A; C4: complement component 4; ApoB: apolipoprotein **b**; ApoA: apolipoprotein A. ^*^Significant differences vs. HDs serum (*p* value< 0.05 and FDR < 0.1)

Although these patients were in remission, serum levels of inflammatory cytokines including TNF-α, IL-6 and IL-1β were significant elevated compared to HDs (Fig. 2a-c). Of note, serum levels of adipokines were altered in JIA patients, showed by a significant increase in visfatin and resistin levels, suggesting an alteration in adipose tissue related to JIA (Fig. 2d-g). These results suggest that the longstanding inflammation, even under remission conditions, might promote alterations in the adipose tissue of these patients, increasing the risk of cardiovascular disease. Likewise, levels of molecules closely involved in endothelial dysfunction were elevated in JIA patients with a significant augmentation of VEGF-A, indicating an activation of the vascular endothelium (Fig. 2h-j).

In addition, levels of all those molecules correlated with clinical parameters. Thus, levels of inflammatory cytokines (IL-1β, TNF-α and IL-6) significantly correlated with clinical inflammatory markers such as C4 and with the duration of the disease, independently on the remission state (Fig. 2k). On the other hand, increased levels of adipokines such as visfatin significantly correlated with altered levels of ApoB and ApoA, suggesting its contribution to lipid metabolism. In addition, augmented resistin levels were in line with the increase in the TNF-α, indicating the relationship between inflammation and adipose tissue dysfunction in JIA patients (Fig. 2k).

**Fig. 3:**
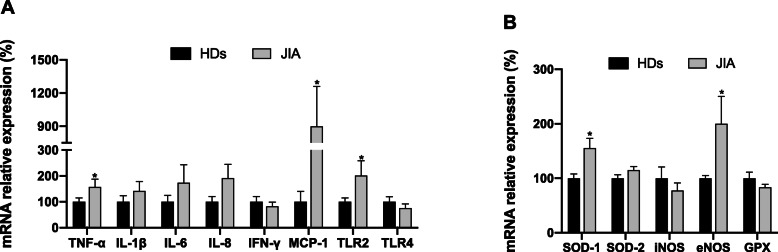
Alterations in the expression of pro-inflammatory and oxidative stress genes in PBMCs of JIA patients compared to HDs. **a** Inflammatory mediators. **b** Oxidative stress markers. TNF-α: tumor necrosis factor-alpha; IL-1β: interleukin-1β; IL-6: interleukin-6; IL-8: interleukin-8; IFN-γ: interferon-γ; MCP-1: monocyte chemoattractant protein-1; TLR-2: toll like receptor-2; TLR-4: toll like receptor-4. ^*^Significant differences vs. HDs (*p* value < 0.05 and FDR < 0.1)

Moreover, peripheral mononuclear cells of JIA patients (monocytes and lymphocytes) had increased expression of inflammatory mediators, such as TNF-α, IL-8, MCP-1 and TLR-2 (Fig. 3a), and oxidative stress markers including SOD-1 and eNOS (Fig. 3b).

Since 60% out of the JIA patients were under treatments, we analyzed whether those treatments could interfere in all the parameters studied. No statistical differences were observed in the prevalence of CV risk factors and the levels of inflammatory mediators, adipokines and endothelial dysfunction and oxidative stress markers between patients in remission with no treatments and with any treatment.

### JIA serum induces alterations in peripheral mononuclear and endothelial cells and adipocytes

**Fig. 4:**
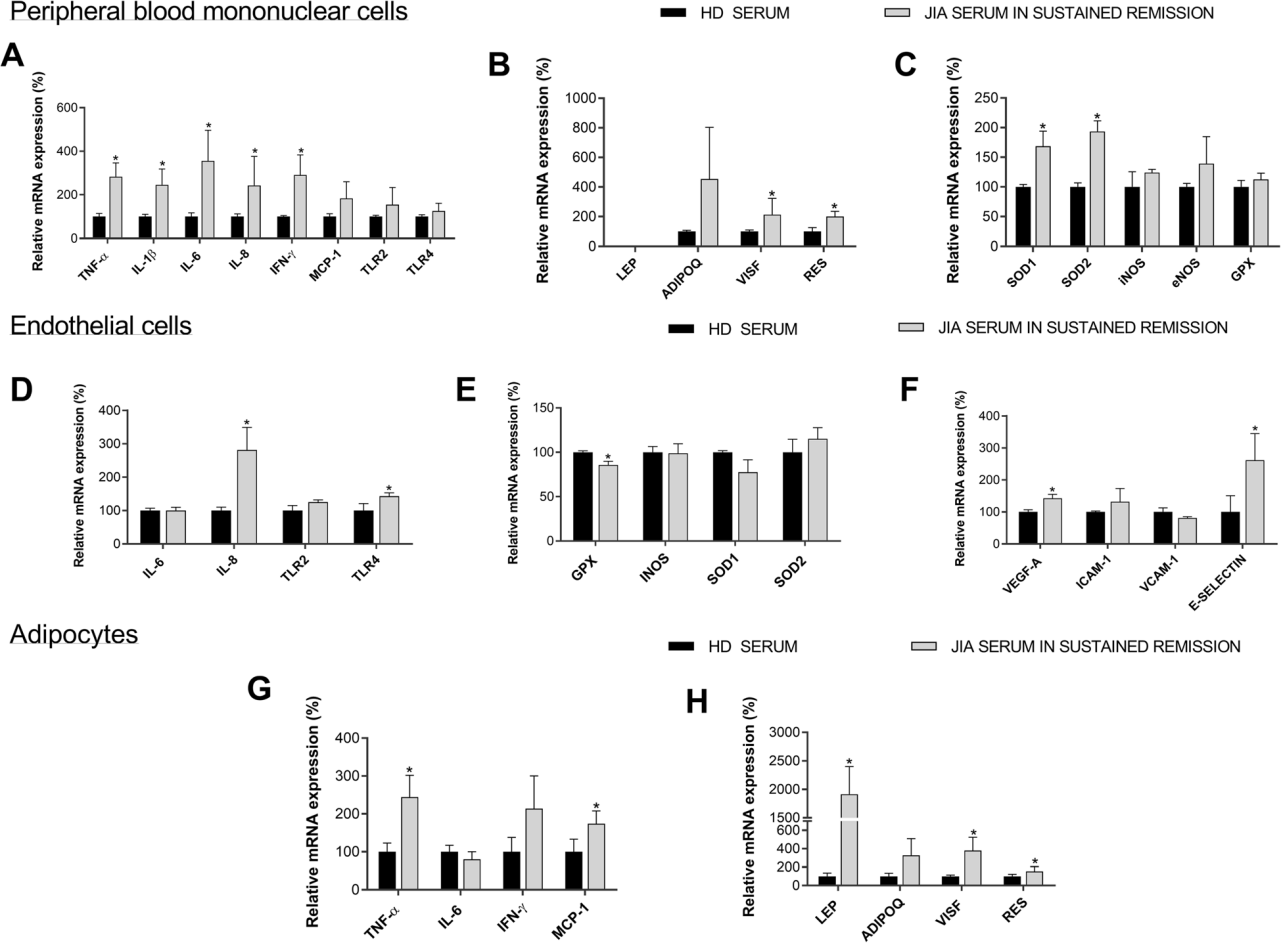
Serum from JIA adult patients in remission induces alterations in PBMCs, endothelial cells and adipocytes compared to HDs serum. **a** Relative gene expression of inflammatory mediators in PBMCs. **b** Relative gene expression of adipokines in PBMCs. **c** Relative gene expression of oxidative stress markers in PBMCs. **d** Relative gene expression of inflammatory mediators in HUVECs. **e** Relative gene expression of oxidative stress markers in HUVECs. **f** Relative gene expression of adhesion molecules in HUVECs. **g** Relative gene expression of inflammatory mediators in adipocytes. **h** Relative gene expression of adipokines in adipocytes. TNF-α: tumor necrosis factor-α; IL-1β: interleukin-1β; IL-6: interleukin-6; IL-8: interleukin-8; IFN-γ: interferon-γ; MCP-1: monocyte chemoattractant protein-1; TLR-2: toll like receptor-2; TLR-4: toll like receptor-4; LEP: Leptin; ADIPOQ: adiponectin; VISF: visfatin; RES: resistin; SOD-1: superoxide dismutase-1; SOD-2: superoxide dismutase-2; iNOS: inducible nitric oxide synthase; eNOS: endothelial nitric oxide synthase; GPX-1: glutathione peroxidase-1; VEGF-A: vascular endothelial growth factor-A. ^*^Significant differences vs. HDs serum (*p* < 0.05)

The effect of serum from those JIA patients in remission, which has been shown to have high levels of inflammatory mediators, on healthy cells was analyzed. The treatment of peripheral mononuclear cells isolated from healthy donors with serum from JIA patients in remission promoted an increase in the levels of inflammatory mediators (TNF-α, IL-1β, IL-6, IL-8 and IFN-γ), oxidative stress markers (SOD-1 and SOD-2) and adipokines (visfatin and resistin) compared to the treatment with serum from healthy donors (Fig. 4a-c).

In endothelial cells, the treatment with JIA serum induced the mRNA expression of VEGF-A and E-selectin, and inflammatory mediators such as IL-8 and TLR-4, suggesting that this serum is able to activate the endothelial cells (Fig. 4d-f).

As altered levels of adipokines on the serum of JIA patients was observed, we evaluated the direct effect of the JIA serum in the adipocytes. Thus, the treatment of these cells with serum of JIA patients induced the expression of inflammatory cytokines and chemokines, and significantly elevated the expression of adipokines, such as leptin, visfatin and resistin, compared to the treatment with the serum from healthy donors, resembling to what was observed in vivo in patients (Fig. 4g, h).

## Discussion

As a long lasting chronic inflammatory disease, concern has been raised regarding the risk of premature development of CVD in JIA. Several studies have been carried out to evaluate the CVD risk related to this pathology, most of them performed in children and adolescents, not considering a long remission state. This study describes for the first time that young adult JIA patients in clinical remission with or without treatment does not show increased traditional CVD risk factors nor early atherosclerosis, however do display subclinical signs of enhanced CVD risk, including high levels of inflammatory cytokines, adipokines, oxidative stress and endothelial activation markers, molecules with a relevant role in the onset and progression of endothelial dysfunction and atherosclerosis.

Obesity, MetSyn and smoking are recognized risk factors for CVD. Evidence regarding obesity rates in JIA is conflicting. Several studies described low BMI in patients with JIA, while others reported no differences with the general population or even increased BMI score in patients with JIA (reviewed in Coulson et al., 2013) [5]. In our cohort of adults JIA patients in remission, BMI was similar in JIA patients and HDs, which means that further studies on the effect of long-term disease and activity in BMI in patients should be carried out. On the other hand, there are few studies evaluating the prevalence of MetSyn or its hallmark, insulin resistance, in adults with JIA, and more specifically being under remission state. In our cohort of JIA patients with a duration of the disease of 13.47 ± 5.47 years, there were similar rates of MetSyn and insulin resistance between patients and age and sex-matched healthy donors. A recent study has evaluated the risk of MetSyn in adults with a history of juvenile arthritis, finding increased rates of MetSyn in this population compared to a non-arthritis cohort [30].

There are few studies reporting smoking rates in adolescents or adults with JIA. So far, published data reported same or even less smoking habit in young people with JIA [5]. To our knowledge this is the first study that reports rates of tobacco use in JIA adults, and similarly to what those studies found, no differences in the smoking habit were observed in our cohort of JIA adults.

A number of publications have informed some data about lipid profile in children with JIA, although the results are controversial. Several authors reported decreased levels of HDL and higher levels of triglycerides in children with JIA, although these observations were more associated with high disease activity (reviewed in [5]). Our data shows that adults JIA patients had significantly increased levels of TC even though they were on a remission state, suggesting that long duration disease could affect lipid profile regardless the disease activity.

In addition, early atherosclerosis has been studied in JIA [31]. Results are conflicting since several cross-sectional studies described greater CIMT in patients with oligo- and polyarticular JIA [32, 33], while others pointed out to similar CIMT between patients and controls [34]. None of these studies took into account patients in remission. In our cohort of adult JIA patients, a trend to increased CIMT was observed with no significant differences, which might suggest that abnormalities in CIMT could be likely given to a longer active disease duration.

Of note, in our cohort of JIA patients elevated serum levels of inflammatory cytokines including TNF-α, IL-6 and IL-1β are evidenced. Pro-inflammatory cytokines are known to induce the differentiation and activation of inflammatory cells, the activation of osteoclasts and general inflammation at the joint level. In this context, it is not surprising that previous studies reported levels of these pro-inflammatory cytokines related to the disease activity and the response to determined treatments [35]. That makes this finding relevant since in our cohort of JIA patients serum levels of inflammatory cytokines were significantly elevated compared to HDs despite being in clinical remission state. Besides, mRNA expression of inflammatory molecules was elevated in leukocytes purified from those JIA patients. Focusing on persistent inflammation as a driver for the development of CVD risk, these cytokines (IL-1β, IL-6 and TNF-α have been implicated in the development of atherosclerosis in rheumatoid arthritis [36], suggesting that in JIA adults under remission phase still could persist subclinical markers of CVD risk which should be monitored.

In addition, the high levels of pro-inflammatory molecules observed in active JIA were associated with endothelial activation [37]. In this sense, serum levels of VEGF-A were also significantly elevated in our cohort of JIA patients in clinical remission, alongside an increase in the mRNA expression of molecules involved in oxidative stress and endothelial activation of JIA PBMCs, suggesting an alteration in the endothelium. Data that was confirmed with the fact that the treatment of healthy PBMCs and endothelial cells with serum from those JIA patients induced the expression of inflammatory, oxidative stress and endothelial activation molecules, indicating that molecules present in the serum of those patients could activate the vascular system even though they were in clinical remission.

On the other hand, adipose tissue has recently been recognized as a complex and dynamic endocrine organ with an intricate role in homeostasis of the whole body [38, 39]. Thus, adipokines are involved in not only in lipid and glucose metabolism, but also cardiovascular homeostasis and inflammatory and immune functions among other physiological functions. In consequence, adipokines such as leptin, resistin and adiponectin are considered key players in the regulation of inflammation [40–42]. In the present work, serum levels of adipokines were altered in JIA patients, showed by a significant increase in the serum levels of resistin and visfatin. In addition, in vitro treatment of PBMCs and adipocytes with serum from JIA patients in sustained remission induced the mRNA expression of inflammatory molecules and these adipocytokines. Similar to what others group have found, in our hands levels of leptin were not different between JIA patients and controls [43, 44]. In addition, in refractory JIA children, no alteration in adiponectin levels was found, which also is in line with our data [45]. On the other hand, resistin levels has already been shown elevated in children with JIA compared to the control group, regardless BMI and were dependent on the disease activity [46]. However, no study has evaluated the levels of resistin in adults JIA patients. Contrary to what those authors reported, our study revealed high levels of resistin in remission state. Moreover, high levels of visfatin has been described in JIA and shown to be a potential biomarker of Methotrexate response in JIA children [47]. Both adipokines, visfatin and resistin have an established proinflammatory role, the fact that these molecules were elevated in our cohort of JIA patients supports the concept that subclinical inflammation might exist in JIA under a long time of remission.

These results might indicate that the longstanding inflammation, even under remission conditions, might promote alterations in the adipose tissue of these patients, contributing to a subclinical cardiovascular risk in these patients.

The major limitation of this study lies in the limited number of patients involved. The small n size did not allow us to stratify patients according to JIA subtypes, presence of CVD risk factors or different treatments received. Meanwhile changes in parameters such as the increase in the CIMT in JIA can be significant in studies including a larger number of patients, we did not find any significant differences among JIA patients and HDs in our study. This fact might be due to the remission state or the number of patients included instead. Studies with larger sample size should be carried out to strengthen our conclusions. The study design (cross-sectional) could be another limitation, since we are not able to detect changes in the clinical and subclinical cardiovascular risk signs possible occurred in the remission state compared to the active phase in the same patient.

## Conclusions

Long-term JIA adult patients in remission might have subclinical signs of inflammation and CVD risk, showed by an increase in the levels of inflammatory cytokines, endothelial activation and oxidative stress markers and adipokines, molecules closely involved in the alteration of the vascular system.

## Data Availability

The datasets used and/or analyzed during the current study are available from the corresponding author on reasonable request.

## Abbreviations

JIA: Juvenile Idiopathic Arthritis
CVD: Cardiovascular Disease
RA: Rheumatoid arthritis
cIMT: Carotid intima media thickness
IL-1: Interleukin 1
IL-6: Interleukin 6
TNF-α: Tumor necrosis factor alpha
ICAM: Intercellular adhesion molecule
ILAR: International League of Associations for Rheumatology
JADAS: Juvenile Arthritis Disease Activity Score
CRP: C reactive protein
ACPAs: Anti-citrullinated protein antibodies
RF: Rheumatoid factor
Hb1Ac: Hemoglobin 1Ac
TC: Total cholesterol
HDL: High density lipoprotein; Triglycerides (TGs)
ApoA: Apolipoprotein A
ApoB: Apolipoprotein B
ESR: Erythrocyte sedimentation rate
BMI: Body Mass Index
T2DM: Type 2 Diabetes Mellitus
MetSyn: Metabolic Syndrome
HOMA-IR: Homeostatic Model Assessment-Insulin Resistance
AdipoQ: Adiponectin
PBMCs: Peripheral Blood Mononuclear Cells
HUVECs: Human Umbilical Vein Endothelial Cells
HD: Healthy Donors
IFN-γ: Interferon-γ
MCP-1: Monocyte chemoattractant protein 1
TLR2: Toll Like Receptor 2
TLR4: Toll Like Receptor 4
SOD-1: Superoxide Dismutase 1
SOD-2: Superoxide Dismutase 2
iNOS: inducible Nitric Oxide Synthase
eNOS: endothelial Nitric Oxide Synthase
GPX-1: Glutathione Peroxidase-1
VEGF-A: Vascular Endothelial Growth Factor A
VCAM: Vascular Cell Adhesion Molecule
RT-PCR: Reverse Transcription Polymerase Chain Reaction
GAPDH: glyceraldehyde-3-phosphate dehydrogenase
HPRT: hypoxanthine-guanine phosphoribosyltransferase
